# Cardiac biomarkers, cardiac injury, and comorbidities associated with severe illness and mortality in coronavirus disease 2019 (COVID‐19): A systematic review and meta‐analysis

**DOI:** 10.1002/iid3.471

**Published:** 2021-08-18

**Authors:** Zhengchuan Zhu, Miaoran Wang, Wei Lin, Qiaoyan Cai, Ling Zhang, Daxin Chen, Fei Liu, Xiaoman Xiong, Jianfeng Chu, Jun Peng, Keji Chen

**Affiliations:** ^1^ Peking University Traditional Chinese Medicine Clinical Medical School (Xiyuan) Beijing China; ^2^ Xiyuan Hospital China Academy of Chinese Medical Sciences Beijing China; ^3^ Academy of Integrative Medicine Fujian University of Traditional Chinese Medicine Fuzhou Fujian China; ^4^ Fujian Key Laboratory of Integrative Medicine on Geriatrics Fujian University of Traditional Chinese Medicine Fuzhou Fujian China; ^5^ Chen Keji Academic Thought Heritage Studio Fujian University of Traditional Chinese Medicine Fuzhou Fujian China; ^6^ National Clinical Research Center for Chinese Medicine Cardiology Beijing China

**Keywords:** acute myocardial injury, cardiac biomarkers, comorbidities, COVID‐19, meta‐analysis, SARS‐CoV‐2

## Abstract

**Aims:**

To explore the correlation between cardiac‐related comorbidities, cardiac biomarkers, acute myocardial injury, and severity level, outcomes in COVID‐19 patients.

**Method:**

Pubmed, Web of Science, Embase, CNKI, VIP, Wanfang, Cochrane Library databases, medRxiv, and Sinomed were reviewed systemically. Various types of clinical research reporting cardiac‐related comorbidities, cardiac biomarkers including lactate dehydrogenase (LDH), troponin I (TnI), high sensitivity troponin I (hs‐TnI), creatine kinase (CK), creatine kinase–MB (CK‐MB), myoglobin (Myo), N‐terminal pro‐b‐type natriuretic peptide (NT‐proBNP) and acute cardiac injury grouped by severity of COVID‐19 were included. Outcome measures were events and total sample size for comorbidities, acute cardiac injury, and laboratory parameters of these biomarkers. The study was performed with Stata version 15.1.

**Results:**

Seventy studies, with a total of 15,354 cases were identified. The results showed that COVID‐19's severity was related to cardiovascular disease. Similar odds ratios (ORs) were achieved in hypertension except for severe versus critical group (OR = 1.406; 95% CI, 0.942–2.097; *p* = .095). The relative risk (RR) of acute cardiac injury is 7.01 (95% CI, 5.64–8.71) in non‐survivor cases. When compared with the different severity of cardiac biomarkers, the pool OR of CK, CK‐MB, TnI, Myo and LDH were 2.683 (95% CI, 0.83–8.671; *p* = .106; *I* ^2^ = 0%), 2.263 (95% CI, 0.939–5.457; *p* = .069), 1.242 (95% CI, 0.628–2.457; *p* = .534), 1.756 (95% CI, 0.608–5.071; *p* = .298; *I* ^2^ = 42.3%), 1.387 (95% CI, 0.707–2.721;  *p* = .341; *I* ^2^ = 0%) in the critical versus severe group, whose trends were not similar to other groups. The standard mean differences (SMD) of CK and TnI in the critical versus severe group were 0.09 (95% CI, −0.33 to 0.50; *p* = .685; *I* ^2^ = 65.2%), 0.478 (95% CI, −0.183 to 1.138; *p* = .156; *I* ^2^ = 76.7%), which means no difference was observed in the serum level of these indicators.

**Conclusion:**

Most of the findings clearly indicate that hypertension, cardiovascular disease, acute cardiac injury, and related laboratory indicators are associated with the severity of COVID‐19. What is now needed are cross‐national prospectively designed observational or clinical trials that will help improve the certainty of the available evidence and treatment decisions for patients.

## Graphical abstract

We use a meta‐analysis to explore the correlation between cardiac‐related comorbidities, cardiac biomarkers, acute myocardial injury, and severity level, outcomes in patients with COVID‐19. Most of the findings clearly indicate that hypertension, cardiovascular disease, acute cardiac injury, and related laboratory indicators are associated with the severity of COVID‐19. What is now needed are cross‐national prospectively designed observational or clinical trials that will help improve the certainty of the available evidence and treatment decision for patients.

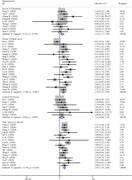

## 1 INTRODUCTION

Coronavirus disease 2019 (COVID‐19), a severe respiratory disease, has caused an unprecedented pandemic crisis. As of August 1, 2020, a total of 17,396,943 cases of COVID‐19 infected by severe acute respiratory syndrome coronavirus 2 (SARS‐CoV‐2), 675,060 deaths have been confirmed by the WHO, and nearly 300 thousand new cases were reported in every 24 h all around the world. SARS‐CoV‐2 is approximately 80% gene sequence similarity to SARS‐CoV,^1^, ^2^, ^3^ which infected host human cells by binding to the receptor proteins, known as angiotensin‐converting enzyme 2 (ACE2) receptor. And the disease transmission eventually contributed to a pneumonia epidemic outbreak in 2003.^4^, ^5^ ACE2 receptor is highly expressed in multiple organ systems^6^ and plays an essential negative role in the ACE‐angiotensin II (Ang II)‐angiotensin II receptor type 1 (AT1R) pathway called the classical renin‐angiotensin‐aldosterone system (RAAS) axis, whose positive effect can increase sympathetic nervous system tension.^7^

According to the current observation, it is the cardiovascular complications and myocardial injury that should be paid close attention to, while most of the attention has been paid to the pulmonary system. The relationship between coronavirus disease 2019 and clinical characters has been much more precise. Firstly cardiovascular diseases, including coronary heart disease and hypertension, can increase the risks for adverse outcomes such as the rate of critical situations and death.^8^ Secondly, a notable proportion of patients experience cardiovascular symptoms, and myocardial injury indicators change at the initial presentation. Several recent studies have described the clinical characteristics and epidemiological findings of COVID‐19 that were tightly implicated in the cardiovascular system.^9^, ^10^, ^11^ Nevertheless, due to the mounting clinical research, further and broader elucidation can be carried out. Here we summarize the current literature to explore the correlation between cardiac‐related comorbidities, cardiac biomarkers, acute myocardial injury, and severity level or outcomes in COVID‐19 patients.

## 2 METHODS

### 2.1 Search strategy

Our review followed the Preferred Reporting Items for Systematic Reviews and Meta‐Analyses (PRISMA) recommendations and criteria.^12^ We conducted a literature retrieval by two independent reviewers without language restrictions (Cai Q and Zhang L) in Pubmed, Web of Science, Embase, CNKI, Wanfang, Cochrane Library databases, VIP, medRxiv, and Sinomed using “SARS‐CoV⁃2,” “COVID‐19,” and "2019⁃nCoV" from December 1, 2019, to June 27, 2020. All relevant research should report the outcome including cardiac‐related comorbidities and cardiac biomarkers, including lactate dehydrogenase (LDH), troponin I (TnI), high sensitivity troponin I (hs‐TnI), creatine kinase (CK), creatine kinase–MB (CK‐MB), myoglobin (Myo), N‐terminal pro‐b‐type natriuretic peptide (NT‐proBNP) or acute cardiac injury. Additionally, extra searches were performed in the title or abstract that reported “clinical characteristics” or “clinical data.” Studies that report cardiac biomarkers or acute cardiac injury but did not classify by patients' severity level or outcomes of COVID‐19 were excluded.

### 2.2 Selection criteria

Two independent reviewers (Zhengchuan Zhu and Miaoran Wang) scrutinized all titles and abstracts excluded irrelevant studies. Then they independently reviewed the full reports. Studies were eligible for inclusion if they met the following conditions: (1) study types: retrospective, prospective, cross‐sectional, observational, descriptive or case‐control studies which report cardiac‐related comorbidities, cardiac biomarkers (including CK, CK‐MB, TnI, Myo or NT‐proBNP, LDH) or patient suffered from acute cardiac injury with COVID‐19; (2) patient characteristics: patients should be diagnosed with COVID‐19 and grouped into moderate cases, severe cases or critical cases according to Diagnosis and Treatment Protocol for Novel Coronavirus Pneumonia (trial version 7) from China or WHO interim guidance^13^; (3) interventions: the study should include at least one cohort data for severe versus non‐severe, severe versus moderate, severe versus non‐severe, severe versus critical, or non‐survivor versus survivor cohorts; and (4) outcome: the mean (standard deviation SD) or median (interquartile range; IQR) for each laboratory parameters of these biomarkers should be involved as continuous outcomes. And the event and total sample size for comorbidities and acute cardiac injury should be involved as dichotomous outcomes.

The exclusion criteria were: (1) study types: case reports, reviews, editorial materials, conference abstracts, and summaries of discussions, (2) patient characteristics: patients in a specific condition such as those who were pregnant (3) recruiters were infected by other coronaviruses, including the severe acute respiratory syndrome (SARS) or the Middle East respiratory syndrome (MERS) human coronavirus or (4) study with insufficient data information in the event.

### 2.3 Data extraction

Independent reviewers (Qiaoyan Cai, Ling Zhang) extracted data into a predesigned sheet and the results were checked by each reviewer. In case of disagreements, a consensus was reached through discussion or another reviewer (Zhengchuan Zhu) examination. Extracted data included: the name of the author, date of publication for the articles, name of the journal, study design, sample size, the diagnostic criteria, the severity of the disease, cardiac‐related comorbidities, and cardiac biomarkers. Mean and standard deviation of indicator values are calculated from the median, interquartile range, and sample size, through Hong Kong Baptist University's website (http://www.math.hkbu.edu.hk/%7Etongt/papers/median2mean.html). The Newcastle‐Ottawa Scale (NOS) quality assessment scale was utilized by two investigators (Zhengchuan Zhu and Miaoran Wang) to assess the quality of studies included in the meta‐analysis. According to these criteria, each study can receive a maximum of nine points. Studies with NOS scores of ≥5 are considered to be high‐quality studies in this systematic review and meta‐analysis.

### 2.4 Statistical analysis

All patients were divided into four cohorts before conducting the study: severe versus moderate, severe versus critical, severe versus non‐severe, and survivor versus non‐survivor based on the diagnosis of COVID‐19 in every study. To reduce the influence from different detection methods, dichotomous outcomes (e.g., cardiac‐related comorbidities) were extracted as odds ratio (OR) with 95% confidence interval (CI), continuous outcomes were extracted as standard mean difference (SMD) with 95% CI. Summary relative risks (RRs) with 95% CI were carried out for the association between acute cardiac injury and the severity of COVID‐19. Heterogeneity among the included studies was assessed using the Cochran's *Q* test (*p* < .05) and the I^2^ statistic(considered *I* ^2^ values <25% to represent low heterogeneity, 25%–50% to moderate heterogeneity, and >50% to severe heterogeneity). The fixed effects models were applied when *I* ^2^ ≤ 50% or *p* ≥ .05, while the random effect models were used by the Mantel‐Haenszel method. To probe the sources of heterogeneity, we employed a leave‐one‐out analysis when *I* ^2^  >  50%. We generated funnel plots and Egger's test to examine the possibility of publication bias. Egger's test with *p* > .05 was considered as funnel symmetry and no publication bias. The statistical analysis was performed with Stata version 15.1 (Stata Corp LP) and GraphPad Prism 7.0 (GraphPad Software).

## 3 RESULTS

### 3.1 Included studies

**Figure 1:**
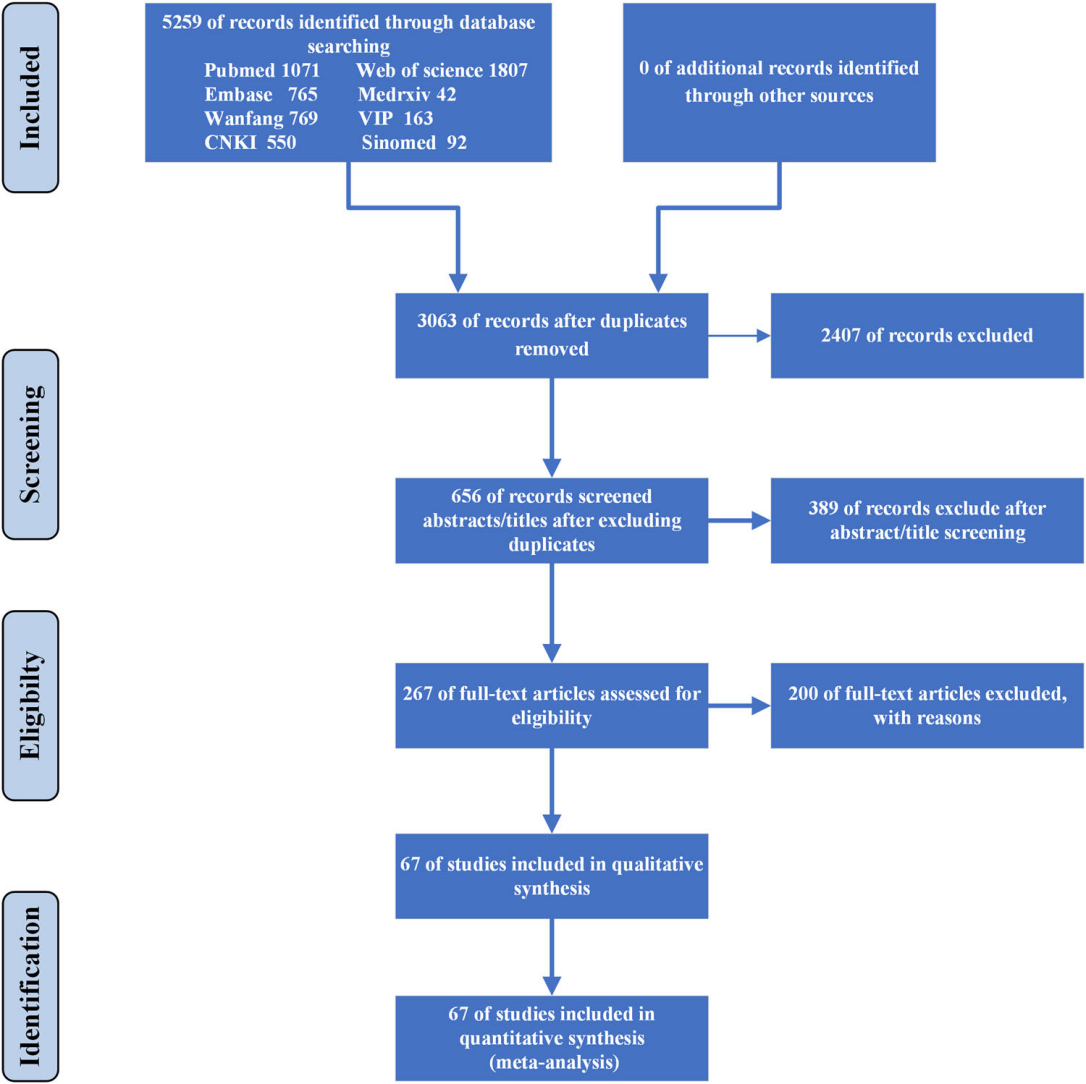
Preferred Reporting Items for Systematic Reviews and Meta‐Analyses (PRISMA) flow chart of the systematic literature review and article identification

Detailed steps of the literature search process is shown in Figure 1. In total, 5259 potentially relevant publications were found from different databases in the initial search. After removing duplications, 3063 were carefully screened, and 2407 unrelated research were removed. And 389 of them were further excluded after screening titles and abstracts. Relevant articles were further selected by reading the full texts. Among these studies, 102 did not report the cardia injury biomarkers, 61 were not classified by the severity of COVID‐19 cases, 14 did not include clear diagnostic criteria, 14 investigated the pregnant and infant cases, 3 were not considered suitable because of their study design, and one suspected considerable overlapping of patients populations in the same hospital. Finally, 67 articles were included.

### 3.2 Baseline characteristics and risk of bias

In total, 67 trails are summarized in the meta‐analysis, and their characteristics are presented in Table 1. A total of 14901 hospitalized patients from four countries were studied in 2020 and the average sample size was 222, which varied from 21 to 1449. The overall average age was greater than 40 years ranged from 1 year to 96 years old and slightly more than half of the sample population was male (51.4%) between 35.48%^22^ to 80.9%.^66^ The most general cardiac‐related comorbidities were coronary heart disease and hypertension. The quality assessment for the included studies is shown in Table 2, and all included studies were evaluated to be high quality with a NOS score ≥6. The *P*‐value of Egger's test in funnel plots was shown in Table 3.

**Table 1:** The characteristics of the included studies in this meta‐analysis

Study	Data	Study design	Region and Country	Diagnostic criteria	*n*	Age(mean ± *SD*)/median w (IQR25, 75)	Sex(male)	Group
^14^	2020/01–2020/02	Retrospective, observational study	Beijing, China	Trial 6th version protocol from China	90	53.0 ± ″″16.9	43	Mild cases (*n* = 2)/Moderate cases (*n* = 55)/severe cases (*n* = 22)/critical cases (*n* = 11)
^15^	2020/01/01–2020/02/06	Retrospective, observational study	Wuhan, China	Trial 5th version protocol from China	463	51 (43, 60)	244	Moderate cases (*n* = 282)/severe cases (*n* = 181)
^16^	2020/01/20–2020/02/10	Retrospective, observational study	Guangzhou, China	Trial 7th version protocol from China	66	Mild cases 38.5 (29, 60.3)/moderate cases 56 (38, 63)/severe cases 57 (55.5, 68.5)/critical cases 66 (60, 83)	29	Mild cases (*n* = 22)/moderate cases (*n* = 31)/severe cases (*n* = 8)/critical cases (*n* = 5)
^17^	2020/01/17–2020/02/20	Retrospective, observational study	Wuhan, China	Trial 6th version protocol from China	89	53.0 ± 16.9	41	Mild cases (*n* = 18)/moderate cases (*n* = 40)/severe cases (*n* = 21)/critical cases (*n* = 10)
^18^	2020/01/24–2020/02/23	Retrospective, observational study	Chongqing, China	Trial 6th version protocol from China	223	46.5 ± 16.1	105	Moderate cases (*n* = 192)/severe cases (*n* = 31)
^19^	2020/01/21–2020/03/15	Retrospective, observational study	Wuhan, China	Trial 5th version protocol from China	30	Death cases 63.6 (median)/survivor cases 65 (median)	15	Survivor cases (*n* = 12)/death cases (*n* = 18)
^20^	2020/01/13–2020/02/12	Retrospective, observational study	Wuhan, China	Trial 6th version protocol from China	274	62(44, 70)	171	Deaths (*n* = 113)/recovered patients (*n* = 161)
^21^	2020/01/01–2020/02/21	Retrospective, observational study	Wuhan, China	Trial 6th version protocol from China	225	Deaths 69 (62, 74)/recover cases 40 (33, 57)	124	Deaths (*n* = 106)/recovered cases (*n* = 116)
^22^	2020/01/01–2020/02/18	Retrospective, observational study	Wuhan, China	WHO interim guidance	273	Mild cases 58.95 ± 10.80/severe cases 58.97 ± 14.38/critical cases 57.27 ± 17.25	97	Mild cases (*n* = 198)/severe cases (*n* = 60)/critical cases (*n* = 15)
^23^	2020/01/03–2020/02/24	Retrospective, observational study	Wuhan, China	Trial 7th version protocol from China	54	68 (59.8, 74.3)	34	Death cases (*n* = 26)/survivor cases (*n* = 28)
^24^	2020/01/16–2020/02/25	Retrospective, observational study	Wuhan, China	Trial 5th version protocol from China	95	49 (39.0, 58)	53	Death cases (*n* = 6)/survivor cases (*n* = 89)
^25^	2020/01/05–2020/01/13	Retrospective, observational study	Wuhan, China	Trial 4th version protocol from China	34	Severe cases 63 (58, 69)/critical cases 67 (66, 75)	17	Severe cases (*n* = 26)/critical cases (*n* = 8)
^26^	2020/01/16–2020/02/20	Retrospective, observational study	non‐Wuhan area of Hubei Province, China	Quantitative polymerase chain reaction assay	91	46 (median)	49	Mild cases (*n* = 61)/severe cases (*n* = 30)
^27^	before 2020/01/31	Retrospective, observational study	Wuhan, China	Trial 6th version protocol from China	191	56 (46, 67)	119	Survivors (*n* = 137)/non‐survivors (*n* = 54)
^28^	2020/01/27–2020/02/27	Retrospective, observational study	Henan, China	Trial 5th version protocol from China	29	56 (31.5, 66)	14	Moderate cases (*n* = 8)/severe cases (*n* = 21)
^29^	2020/01/08–2020/02/20	Retrospective, observational study	Wuhan, China	WHO interim guidance	323	61 (23, 91)	166	Non‐severe (*n* = 151)/severe (*n* = 146)/critical (*n* = 26)
^30^	2020/01/01–2020/02/15	Retrospective, observational study	Wuhan, Shanghai, and Anhui, China	Trial 5th version protocol from China	476	53 (40, 64)	271	Moderate (*n* = 352)/severe (*n* = 54)/critical (*n* = 70)
^31^	2020/01/01–2020/02/15	Retrospective, observational study	Wuhan, China	Trial 7th version protocol from China	25	51 (median)	10	Non‐severe (*n* = 16)/severe (*n* = 9)/; alive (*n* = 20)/dead (*n* = 5)
^32^	2020/01/20–2020/02/5	Retrospective, observational study	Wuhan, China	Trial 5th version protocol from China and WHO interim guidance	584	60 (48, 69)	279	Non‐severe (*n* = 279)/Severe (*n* = 269)
^33^	2020/01/20–2020/04/01	Retrospective, observational study	Wuhan, China	WHO interim guidance and trial 7th version protocol from China	1449	57 (42, 66)	733	Alive (*n* = 1327)/died (*n* = 122)
^34^	2020/01/21–2020/02/12	Retrospective, observational study	Beijing, China	Trial 5th version protocol from China	80	53 ± 20	38	Severe (*n* = 27)/non‐severe (*n* = 53)
^35^	2020/01/12–2020/02/20	Retrospective, observational study	Ningbo, China	Trial 6th version protocol from China	127	50.90 ± 15.26	54	Non‐severe cases (*n* = 111)/severe cases (*n* = 16)
^36^	2020/01/02–2020/02/10	Retrospective, observational study	Wuhan, China	WHO interim guidance	221	55 (39.0, 66.5)	108	Severe (*n* = 55)/non‐severe (*n* = 166)
^37^	2020/01/22–2020/02/10	Retrospective, observational study	Jiangsu, China	Trial 5th version protocol from China	202	44.0 (33, 54)	116	Non‐severe patients (*n* = 179)/severe patients (*n* = 23)
^38^	2019/12/11–2020/01/29	Retrospective, observational study	mainland, China	WHO interim guidance	1099	47.0 (35, 58)	637	Non‐severe (*n* = 926)/severe (*n* = 173)
^39^	2020/01/24–2020/02/07	Retrospective, observational study	Chongqing, China	Trial 6th version protocol from China	114	46.05 ± 15.15	56	Mild cases (*n* = 99)/severe cases (*n* = 4)/critical cases (*n* = 11)
^40^	2020/01/20–2020/02/10	Retrospective, observational study	Guangzhou, China	WHO interim guidance	275	49 (34, 62)	128	Non‐severe Patients (*n* = 230)/severe patients (*n* = 45)
^41^	2020/01/21–2020/02/25	Retrospective, observational study	Chongqing, China	Trial 6th version protocol from China	51	60.94 ± 14.87	27	Severe (*n* = 35)/critical cases (*n* = 16)
^42^	2020/01/24–2020/03/01	Retrospective, observational study	Wuhan, China	Trial 6th version protocol from China	382	52.0 ± 14.1	190	Moderate cases (*n* = 285)/severe cases (*n* = 63)/critical cases (*n* = 34)
^43^	2020/01/01–2020/03/22	Retrospective, observational study	Wuhan, China	Trial 7th version protocol from China	102	51.6 ± 19.3	75	Moderate cases (*n* = 63)/severe cases (*n* = 29)/critical cases (*n* = 10)
^44^	2020/03/01–2020/03/30	Retrospective, observational study	Esine, Brescia, Lombardy, Italy	Real‐time reverse transcriptase polymerase chain reaction	144	In‐hospital death 78.0 (64.2, 84.0)/discharged 62.1 (53.0, 72.8)	96	In‐hospital death (*n* = 70)/discharged (*n* = 74)
^45^	2020/01/01–2020/02/15	Retrospective cohort study	Wuhan, China	Not mention	660	55.0 (34.0, 68.0)	295	Survivor (*n* = 578)/non‐survivor (*n* = 82)
^46^	2020/01/01–2020/03/11	Retrospective, observational study	Zhejiang, China	Trial 7th version protocol from China	145	45.3 ± 13.6	79	Non‐severely ill patients (*n* = 102)/severely ill patients (*n* = 43)
^47^	2019/12/30–2020/02/16	Retrospective, observational study	Wuhan, China	Trial 5th version protocol from China	73	58.36 ± 14.31	49	Non‐survivors (*n* = 47)/survivors (n = 26)
^48^	2020/01/17–2020/02/10	Retrospective, observational study	Wuhan, China	Trial 6th version protocol from China	54	41 (31, 51)	28	Died (*N* = 68)/discharged (*N* = 82)
^49^	2020/02/19–2020/04/15	Retrospective, observational study	Daegu, Korea	ARDS Definition Task ForceRanieri VM, Rubenfeld GD, Thompson BT, Ferguson ND, Caldwell E, et al. Acute respiratory distress syndrome: the Berlin definition.	110	56.9 ± 17.0	48	Severe patients (*n* = 23)/non‐severe patients (*n* = 87)
^50^	2020/01/31–2020/03/05	Retrospective, observational study	Wuhan, China	WHO interim guidance	101	51.0 (37, 61)	48	Non‐survivor (*n* = 15)/survivor (*n* = 87)/
^51^	2020/01/10–2020/02/22	Retrospective, observational study	Wuhan, China	Trial 5th version protocol from China	93	51.0 ± 17.5	41	Survivors (*n* = 68)/non‐survivors (*n* = 25)
^52^	2020/01/20–2020/02/10	Retrospective, observational study	Guangzhou, China	Trial 6th version protocol from China	278	48.1 ± 17.0	130	Mild (*n* = 250)/moderate (*n* = 211)/severe (*n* = 28)/critical (*n* = 14)
^53^	2020/01/10–2020/02/15	Retrospective, observational study	Hubei, China	Trial 7th version protocol from China	85	59.4 ± 15.3	45	General cases (*n* = 46)/severe and critical cases (*n* = 39)
^54^	2020/01/31–2020/02/05	Retrospective, observational study	Wuhan, China	Trial 7th version protocol from China	202	63 (51, 70)	88	non‐survival cases (*n* = 169)/survival cases (*n* = 33)
^55^	2020/01/10–2020/03/13	Retrospective, observational study	8 provinces, China	Trial 6th version protocol from China	703	46.1 ± 15.2	382	Discharge cases (*n* = 659)/death cases (*n* = 33)
^56^	2020/01/30–2020/02/11	Retrospective cohort study	Huanggang, China	The WHO interim guidance the American Thoracic Society guidelines for community‐acquired pneumonia	108	52 (37, 58)	43	Non‐severe (*n* = 83)/severe‐alive (*n* = 13)/severe‐dead (*n* = 12)
^57^	2020/01–2020/02	Retrospective cohort study	Beijing, China	Trial 6th version protocol from China	80	51.2 ± 17.5	33	Mild disease (*n* = 56)/severe (*n* = 24)/died (*n* = 3)
^58^	2019/12/24–2020/01/28	Retrospective, observational study	Wuhan, China	Trial 5th version protocol from China	109	52.5 ± 10.8	48	Moderate cases (*n* = 65)/severe and critical cases (*n* = 44)
^59^	2020/01/22–2020/02/18	Retrospective, observational study	Anhui, China	Trial 6th version protocol from China	79	45.1 ± 16.6	45	General cases (*n* = 55)/severe and critical cases (*n* = 24)
^8^	2020/01/24–2020/02/25	Retrospective, observational study	Guangzhou, China	Trial 7th version protocol from China	82	44.8 ± 15.6	48	Moderate cases (*n* = 58)/severe and critical cases (*n* = 24)
^60^	2020/01/20–2020/02/27	Retrospective, observational study	Zhuzhou, China	Trial 5th version protocol from China	80	47.8 ± 19	40	Mild cases and moderate cases (*n* = 63)/severe cases and critical cases (*n* = 17)
^61^	2020/01/20–2020/02/10	Retrospective, observational study	Shanghai, China	Trial 6th version protocol from China	292	49.9 ± 16.3	154	Mild cases (*n* = 271)/severe cases (*n* = 21)
^62^	2020/01/21–2020/02/11	Retrospective, observational study	Beijing, China	Trial 6th version protocol from China	74	52.7 ± 19.1	35	Common cases (*n* = 56)/severe cases (*n* = 9)/critical severe cases (*n* = 9)
^63^	2020/02–2020/03	Retrospective, observational study	Tübingen, Germany	Real‐time reverse transcriptase polymerase chain reaction	123	68 ± 15	77	Non‐survivors (*n* = 16)/survivors (*n* = 107)
^64^	2020/01/01–2020/02/23	Retrospective, observational study	Wuhan, China	Trial 6th version protocol from China	671	63 (50, 72)	322	Death (*n* = 62)/survivors (*n* = 609)
^65^	2020/02/21–2020/03/19	Retrospective, observational study	Milan, Italy	WHO interim guidance	233	61 (50, 72)	161	Survivors (*n* = 185)/non‐survivors (*n* = 48)
^66^	2020/1/27	Retrospective, observational study	Wuhan, China	Trial 6th version protocol from China	21	56 (50.0–65.0)	17	Severe cases (*n* = 11)/moderate cases (*n* = 10)
^67^	2020/01/01–2020/01/30	Retrospective, observational study	Wuhan, China	RT‐PCR	200	Not mention	99	Alive cases (*N* = 166)/dead cases (*N* = 34)
^68^	2020/02/01–2020/02/18	Retrospective, observational study	Wuhan, China	American Thoracic Society guidelines for community acquired pneumonia	47	64.91 (31–87)	26	Non‐severe patients (*N* = 23)/severe patients (*N* = 24)
^69^	2020/01/10–2020/02/13	Retrospective, observational study	Wuhan, China	Trial 5th version protocol from China	204	49 (34–62)	79	Non‐severe (*n* = 135)/severe (*n* = 69)
^70^	2020/01/30–2020/02/25	Retrospective, observational study	Wuhan, China	Trial 4th version protocol from China	403	56 (39–68)	193	Died (*n* = 100)/recovered (*n* = 303)
^71^	2020/01/21–2020/03/02	Retrospective cohort study	Chongqing, China	Trial 5th version protocol from China	84	48 (42.3–62.5)	48	Severe (*n* = 20)/non‐severe (*n* = 64)
^72^	2020/01/17–2020/02/20	Retrospective, observational study	Changsha, China	The severe cases of COVID‐19 were defined according to the following criteria: respiratory rate ≥30/min or oxygen saturation ≤93% or PaO_2_/FiO_2_ ≤ 300 mmHg or mechanical ventilation or intensive care unit or death.	242	45 (1–84)	99	Severe (*n* = 37)/non‐severe (*n* = 205)
^73^	2020/01/25–2020/02/25	Retrospective, observational study	Wuhan, China	WHO interim guideline	344	64 (52–72)	179	Severely and critically ill patients survivors (*n* = 211)/non‐survivors (*n* = 133)
^74^	not mention	Retrospective, observational study	Anhui, China	Trial 5th version protocol from China	167	42.31 (15.29)	95	Non‐severe (*n* = 137)/severe (*n* = 30)
^75^	2020/01/20–2020/02/20	Retrospective, observational study	Yancheng, Fuyang, Wuxi China	WHO interim guideline	280	43.12 ± 19.02	151	Mild‐and moderate type patients (*n* = 197)/severe‐and critically ill type patients (*n* = 83)
^76^	2020/01/17–2020/02/07	Retrospective, observational study	Changsha, China	Trial 5th version protocol from China	161	45 (33.5, 57)	80	Non‐severe (*N* = 131)/severe (*N* = 30)
^77^	2020/01/23–2020/02/08	Retrospective, observational study	Chongqing, China	Trial 5th version protocol from China	135	47 (36, 55)	72	Mild cases (*n* = 95)/severe cases (*n* = 40)
^78^	2020/01/31–2020/02/05	Retrospective, observational study	Wuhan, China	Trial 7th version protocol from China	202	63 (51, 70)	88	Survivors (*n* = 169)/non‐survivors (*n* = 33)
^79^	2020/03/10–2020/03/30	Retrospective, observational study	England	RT‐PCR	95	75 (59, 82)	60	Survivors (*n* = 75)/non‐survivors (*n* = 20)

*Note:* The protocol from China: Diagnosis and Treatment Protocol for Novel Coronavirus Pneumonia (the number of trial version) edited by the National Health Committee General Office; WHO interim guideline:Clinical management of severe acute respiratory infection when Novel coronavirus(nCoV) infection is suspected:2020 interim guidance.

Abbreviations: qPCR, quantitative polymerase chain reaction assay; RT‐PCR, real‐time reverse‐transcriptase polymerase chain reaction.

**Table 2:** NEWCASTLE‐OTTAWA quality assessment scale for cross‐sectional studies

	Selection				Comparability	Exposure			Total score
	Adequate definition of cases	Representativeness of the cases	Selection of controls	Definition of controls	Control for important factor	Ascertainment of exposure	Same method of ascertainment for cases and controls	Nonresponse rate	
^13^	☆	☆	☆		☆	☆	☆	☆	7
^14^	☆	☆	☆		☆	☆	☆	☆	7
^15^	☆	☆	☆		☆	☆	☆	☆	7
^16^	☆	☆	☆		☆	☆	☆	☆	7
^17^	☆	☆	☆		☆	☆	☆	☆	7
^18^	☆	☆	☆		☆	☆	☆		6
^19^	☆	☆	☆		☆	☆	☆	☆	7
^20^	☆	☆	☆		☆	☆	☆	☆	7
^21^	☆	☆	☆		☆	☆	☆	☆	7
^22^	☆	☆	☆		☆	☆	☆	☆	7
^23^	☆	☆	☆		☆	☆	☆	☆	7
^24^	☆	☆	☆		☆	☆	☆	☆	7
^25^	☆	☆	☆		☆	☆	☆	☆	7
^26^	☆	☆	☆		☆	☆	☆	☆	7
^27^	☆	☆	☆		☆	☆	☆	☆	7
^28^	☆	☆	☆		☆	☆	☆	☆	7
^29^	☆	☆	☆		☆	☆	☆	☆	7
^30^	☆	☆	☆		☆	☆	☆	☆	7
^31^	☆	☆	☆		☆	☆	☆	☆	7
^32^	☆	☆	☆		☆	☆	☆	☆	7
^33^	☆	☆	☆		☆	☆	☆	☆	7
^34^	☆	☆	☆		☆	☆	☆	☆	7
^35^	☆	☆	☆		☆	☆	☆	☆	7
^36^	☆	☆	☆		☆	☆	☆	☆	7
^37^	☆	☆	☆		☆	☆	☆	☆	7
^38^	☆	☆	☆		☆	☆	☆	☆	7
^39^	☆	☆	☆		☆	☆	☆	☆	7
^40^	☆	☆	☆		☆	☆	☆	☆	7
^41^	☆	☆	☆		☆	☆	☆	☆	7
^42^	☆	☆	☆		☆	☆	☆	☆	7
^43^	☆	☆	☆		☆	☆	☆	☆	7
^44^	☆	☆	☆		☆	☆	☆	☆	7
^45^	☆	☆	☆		☆	☆	☆	☆	7
^46^	☆	☆	☆		☆	☆	☆	☆	7
^47^	☆	☆	☆		☆	☆	☆	☆	7
^48^	☆	☆	☆		☆	☆	☆	☆	7
^49^	☆	☆	☆		☆	☆	☆	☆	7
^50^	☆	☆	☆		☆	☆	☆	☆	7
^51^	☆	☆	☆		☆	☆	☆	☆	7
^52^	☆	☆	☆		☆	☆	☆	☆	7
^53^	☆	☆	☆		☆	☆	☆	☆	7
^54^	☆	☆	☆		☆	☆	☆	☆	7
^55^	☆	☆	☆		☆	☆	☆	☆	7
^56^	☆	☆	☆		☆	☆	☆	☆	7
^57^	☆	☆	☆		☆	☆	☆	☆	7
^58^	☆	☆	☆		☆	☆	☆	☆	7
^7^	☆	☆	☆		☆	☆	☆	☆	7
^59^	☆	☆	☆		☆	☆	☆	☆	7
^60^	☆	☆	☆		☆	☆	☆	☆	7
^61^	☆	☆	☆		☆	☆	☆	☆	7
^62^	☆	☆	☆		☆	☆	☆	☆	7
^63^	☆	☆	☆		☆	☆	☆	☆	7
^64^	☆	☆	☆		☆	☆	☆	☆	7
^65^	☆	☆	☆		☆	☆	☆	☆	7
^66^	☆	☆	☆		☆	☆	☆	☆	7
^67^	☆	☆	☆		☆	☆	☆	☆	7
^68^	☆	☆	☆		☆	☆	☆	☆	7
^69^	☆	☆	☆		☆	☆	☆	☆	7
^70^	☆	☆	☆		☆	☆	☆	☆	7
^71^	☆	☆	☆		☆	☆	☆	☆	7
^72^	☆	☆	☆		☆	☆	☆	☆	7
^73^	☆	☆	☆		☆	☆	☆	☆	7
^74^	☆	☆	☆		☆	☆	☆	☆	7
^75^	☆	☆	☆		☆	☆	☆	☆	7
^76^	☆	☆	☆		☆	☆	☆	☆	7
^77^	☆	☆	☆		☆	☆	☆	☆	7
^78^	☆	☆	☆		☆	☆	☆	☆	7

**Table 3:** *p* value of Egger's test in funnel plots

	Items	*p* value of Egger's test	
		Dichotomous outcomes	Continuous outcomes
Severe versus moderate	Hypertension	.97	–
	Cardiovascular disease	.137	–
	Acute cardiac damage	–	–
	CK	.405	.571
	CK‐MB	.197	.421
	LDH	.057	.37
	Myo	.12	.709
	TnI	.541	.661
	NT‐BNP	–	–
Severe versus non‐severe	Hypertension	.708	–
	Cardiovascular disease	.109	–
	CK	.091	.068
	CK‐MB	.059	.767
	TnI	.604	.455
	LDH	.255	.322
	Myo	–	.573
	NT‐BNP	–	.156
Severe versus critical	Hypertension	.037	–
	Cardiovascular disease	.079	–
	CK	–	.85
	CK‐MB	.086	.923
	LDH	.602	.354
	TnI	.092	.074
	Myo	–	.627
Non‐survior versus survivor	Hypertension	.986	–
	Cardiovascular disease	.166	–
	TnI	.445	.162
	CK	.419	.052
	CK‐MB	.343	.693
	NT‐BNP	–	.654
	Acute cardiac injury	.46	–
	LDH	.103	.254
	Myo	.793	.088
Severe versus moderate	Hypertension	.97	–
	Cardiovascular disease	.137	–
	Acute cardiac damage	–	–
	CK	.405	.571
	CK‐MB	.197	.421
	LDH	.057	.37
	Myo	.12	.709
	TnI	.541	.661
	NT‐BNP	–	–
Severe versus non‐severe	Hypertension	.708	–
	Cardiovascular disease	.109	–
	CK	.091	.068
	CK‐MB	.059	.767
	TnI	.604	.455
	LDH	.255	.322
	Myo	–	.573
	NT‐BNP	–	.156

### 3.3 Efficacy analysis

#### 3.3.1 Cardiac‐related comorbidities

**Figure 2:**
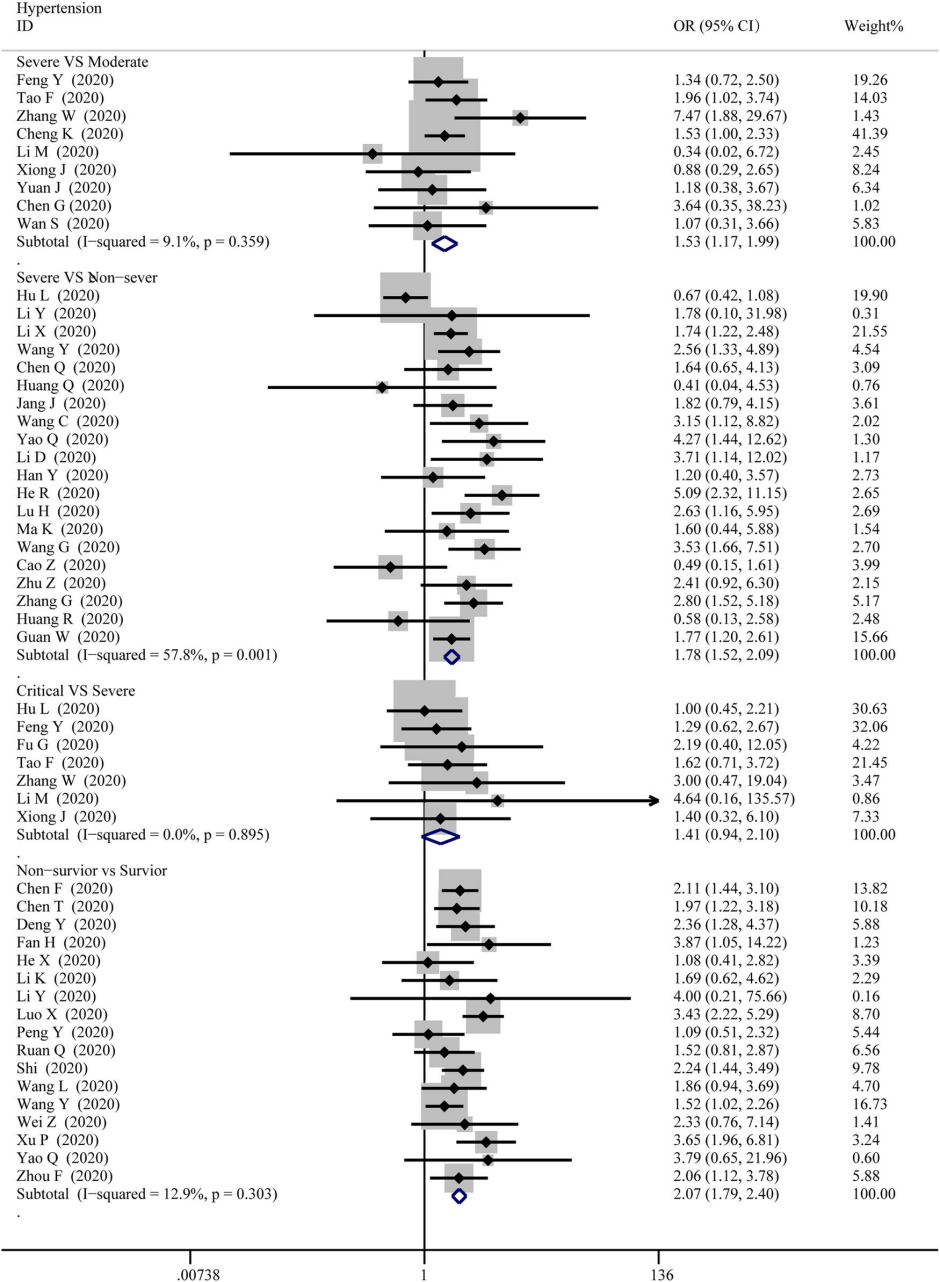
Forest plot comparisons of the number of COVID‐19 cases suffered from hypertension in severe versus moderate, severe versus critical, severe versus non‐severe, and survivor versus non‐survivor group

The data on cardiac‐related comorbidities among patients were extracted from 4 groups, then pooled respectively for meta‐analysis. Figure 2 displays an overview of the number of COVID‐19 cases suffered from hypertension in different severities. The results show that there were marked differences for hypertension between severe and moderate cases (OR = 1.53; 95% CI, 1.17–1.99, *p* = .002; *I* ^2^ = 9.1%), severe and non‐severe cases (OR = 1.78; 95% CI, 1.52–2.09; *p* = 0; *I* ^2^ = 57.8%), non‐survivor and survivor cases (OR = 2.07; 95% CI, 1.79–2.40; *p* = 0; *I* ^2^ = 12.9%), while no differences between critical and severe cases (OR = 1.41; 95% CI, 0.94–2.10, *p* = .00; *I* ^2^ = 0%).

**Figure 3:**
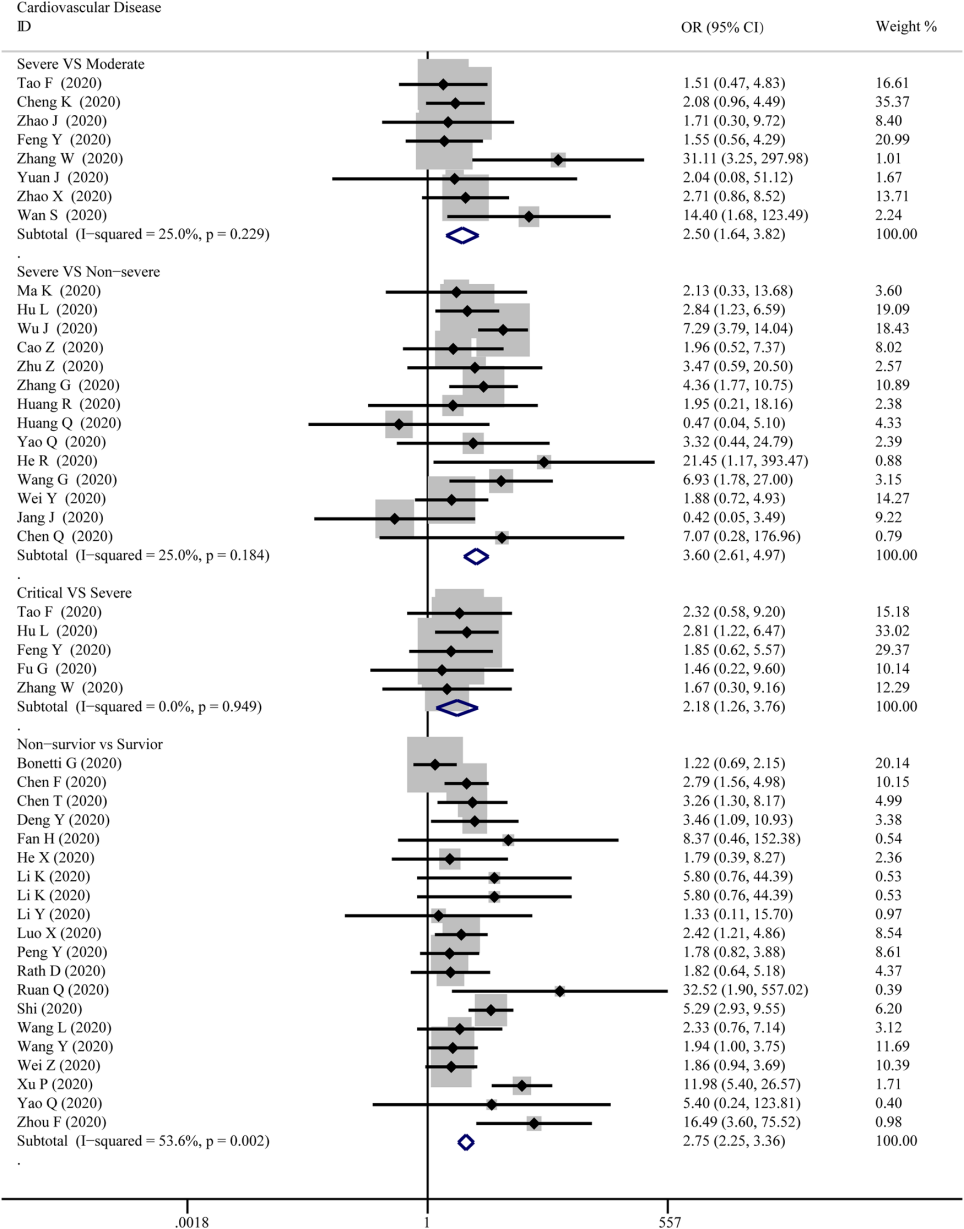
Forest plot comparisons of the number of COVID‐19 cases suffered from cardiovascular disease in severe versus moderate, severe versus critical, severe versus non‐severe, and survivor versus non‐survivor group

As presented in Figure 3, cardiovascular disease was significantly associated with the severity level of COVID‐19 in every group. The combined OR were 2.50 (95% CI, 1.64–3.82; *p* = 0; *I* ^2^ = 25%), 3.60 (95% CI, 2.61–4.97; *p* = 0; *I* ^2^ = 25.0%), 2.18 (95% CI, 1.26–3.76; *p* = .005; *I* ^2^ = 0%), 2.75 (95% CI, 2.25–3.36; *p* = 0; *I* ^2^ = 53.6%), respectively. There were no publishing biases based on the results of Egger's test in funnel plots, except for hypertension in the critical versus severe group (*p* = .037).

#### 3.3.2 Laboratory indicators

**Figure 4:**
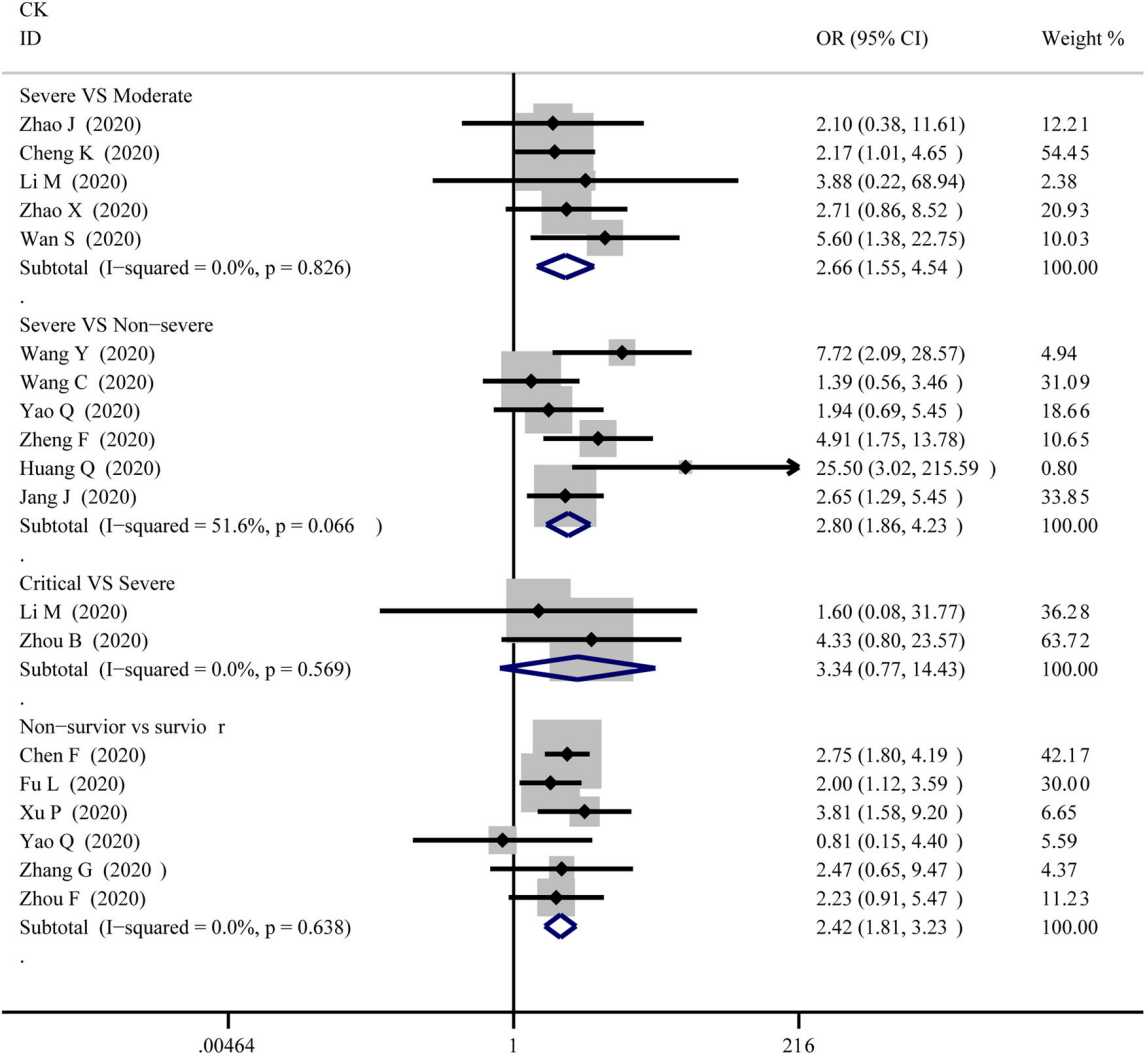
Forest plot comparisons of increasing creatine kinase (CK) in patients with different severity of COVID‐19

**Figure 5:**
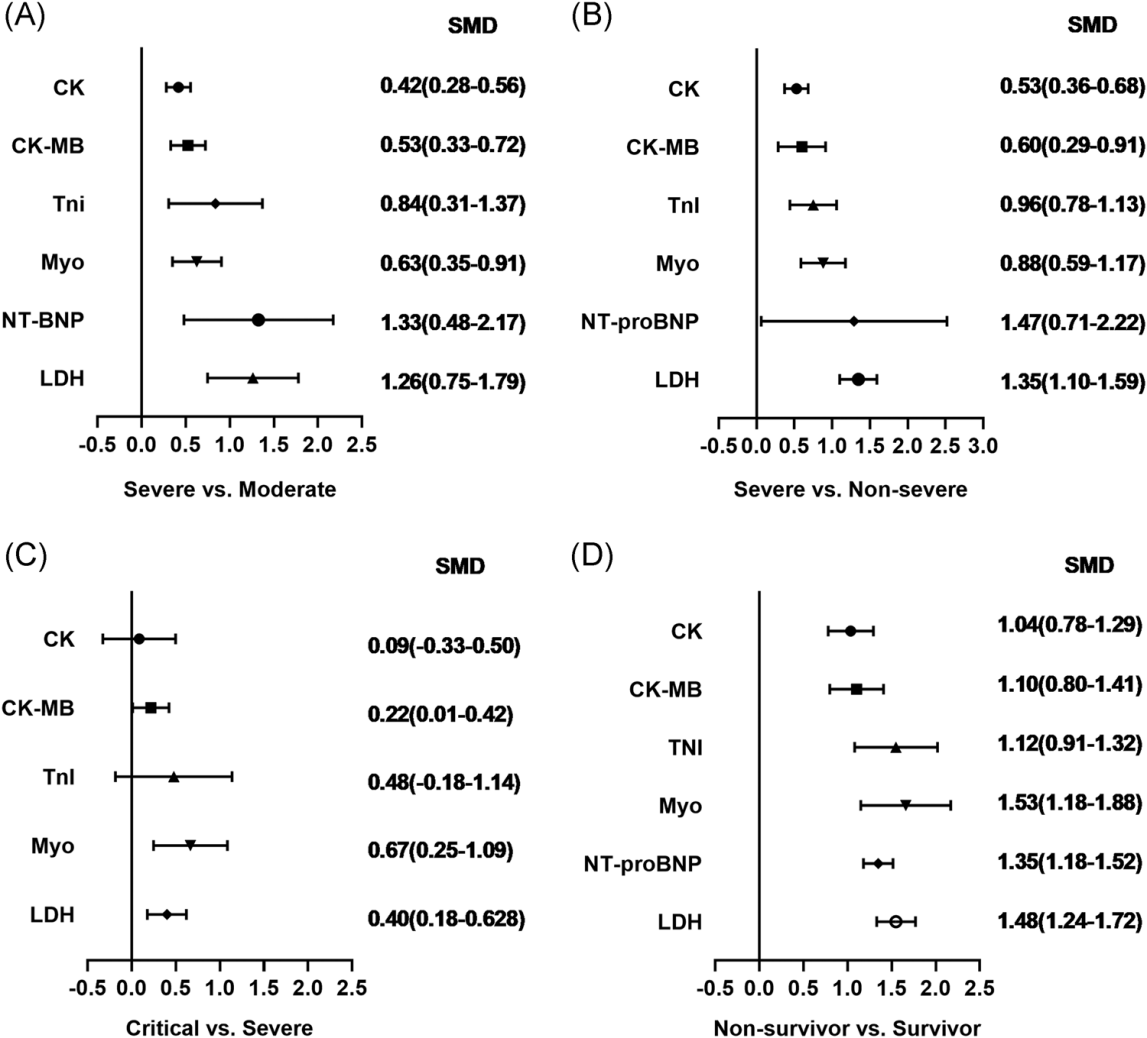
Forest plot comparisons of cardiac biomarkers: (A) severe versus moderate group, (B) severe versus critical group, (C) severe versus non‐severe group, (D) survivor versus non‐survivor group. SMD, standard mean difference

**Figure 6:**
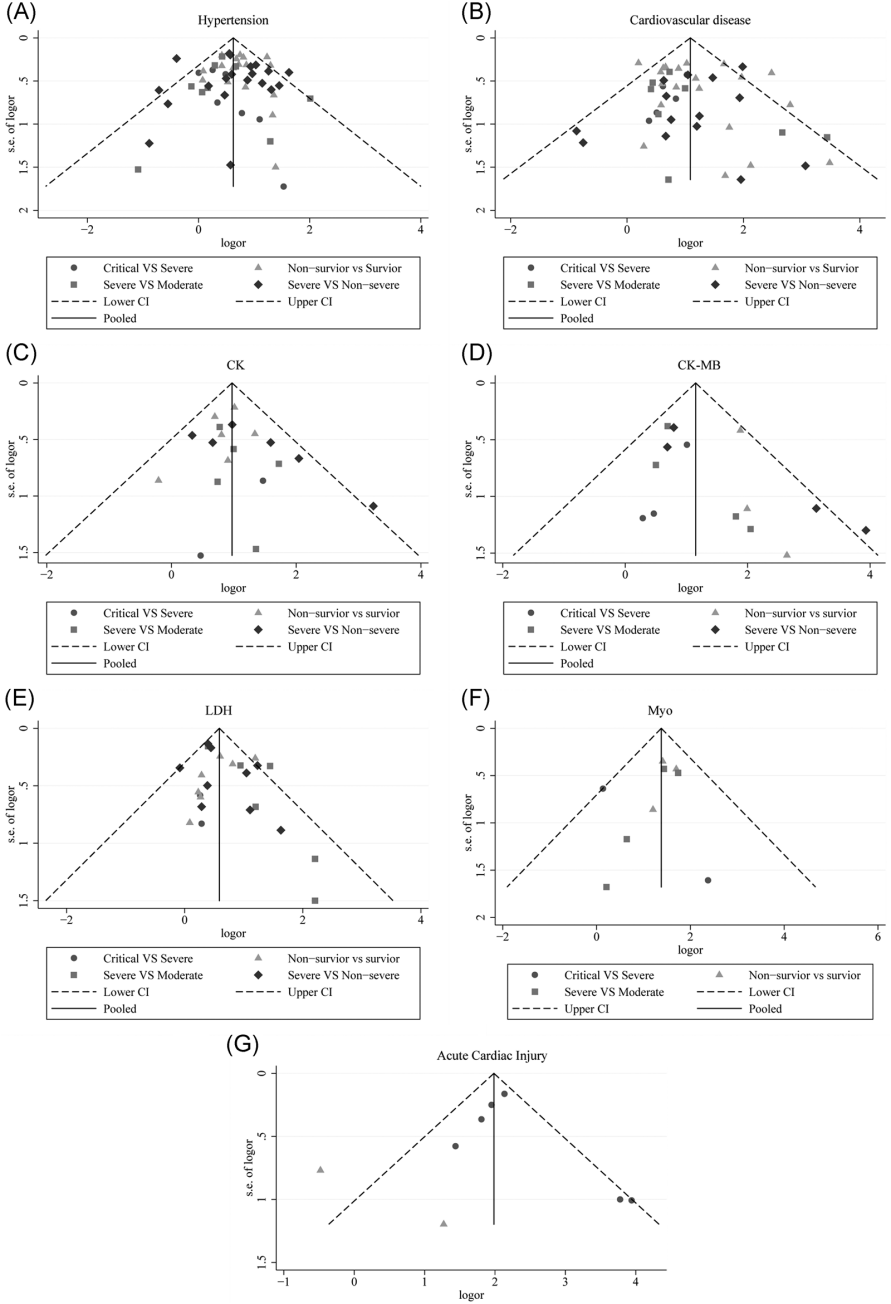
Funnel plot for the assessment of publication bias in dichotomous outcomes: (A) hypertension in different severity and outcome of COVID‐19, (B) cardiovascular disease in different severity and outcome of COVID‐19, (C) CK in different severity and outcome of COVID‐19, (D) CK‐MB in different severity and outcome of COVID‐19, (E) LDH in different severity and outcome of COVID‐19, (F) Myo in different severity and outcome of COVID‐19, (G) TnI in different severity and outcome of COVID‐19

**Figure 7:**
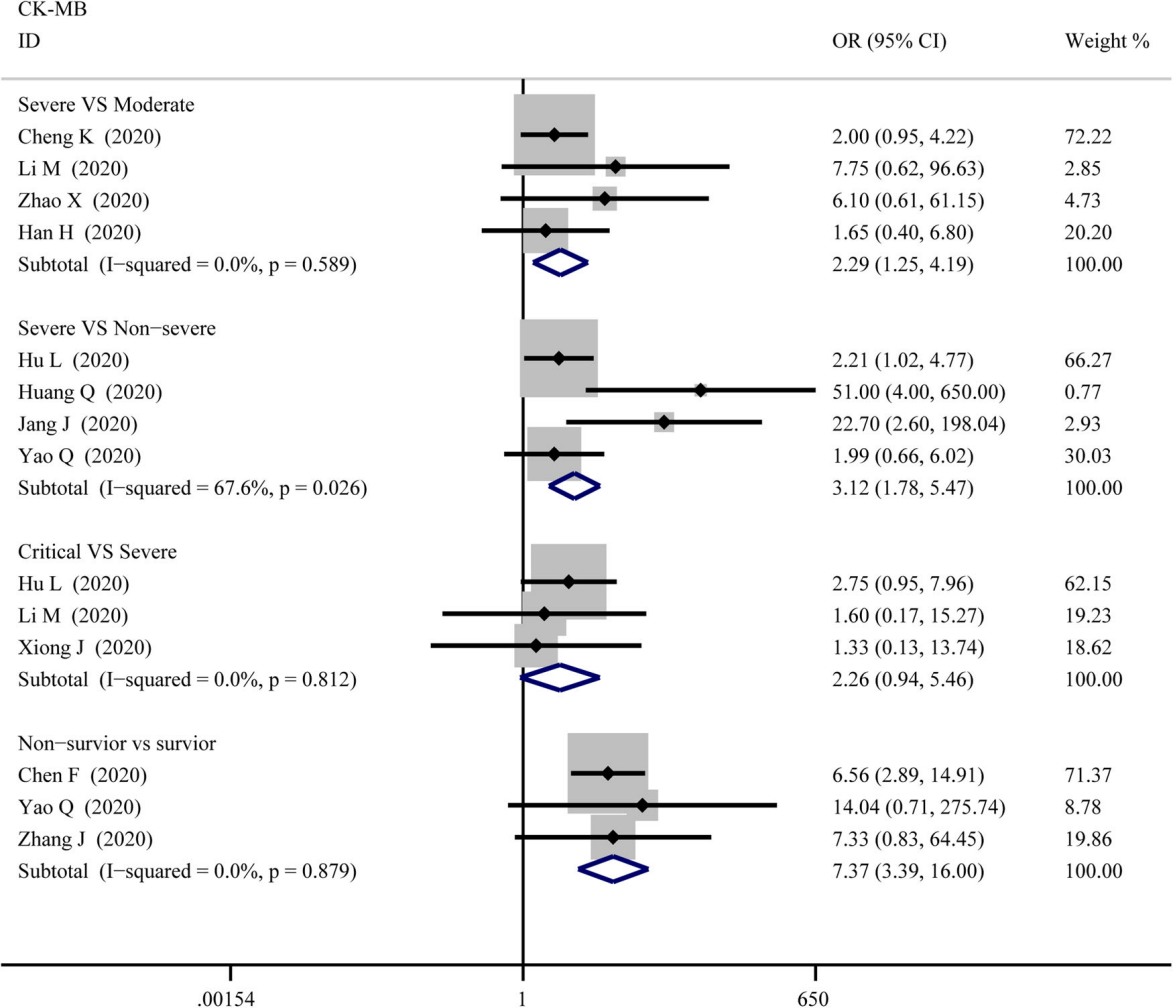
Forest plot comparisons of increasing creatine kinase–MB (CK‐MB) in patients with different severity of COVID‐19

**Figure 8:**
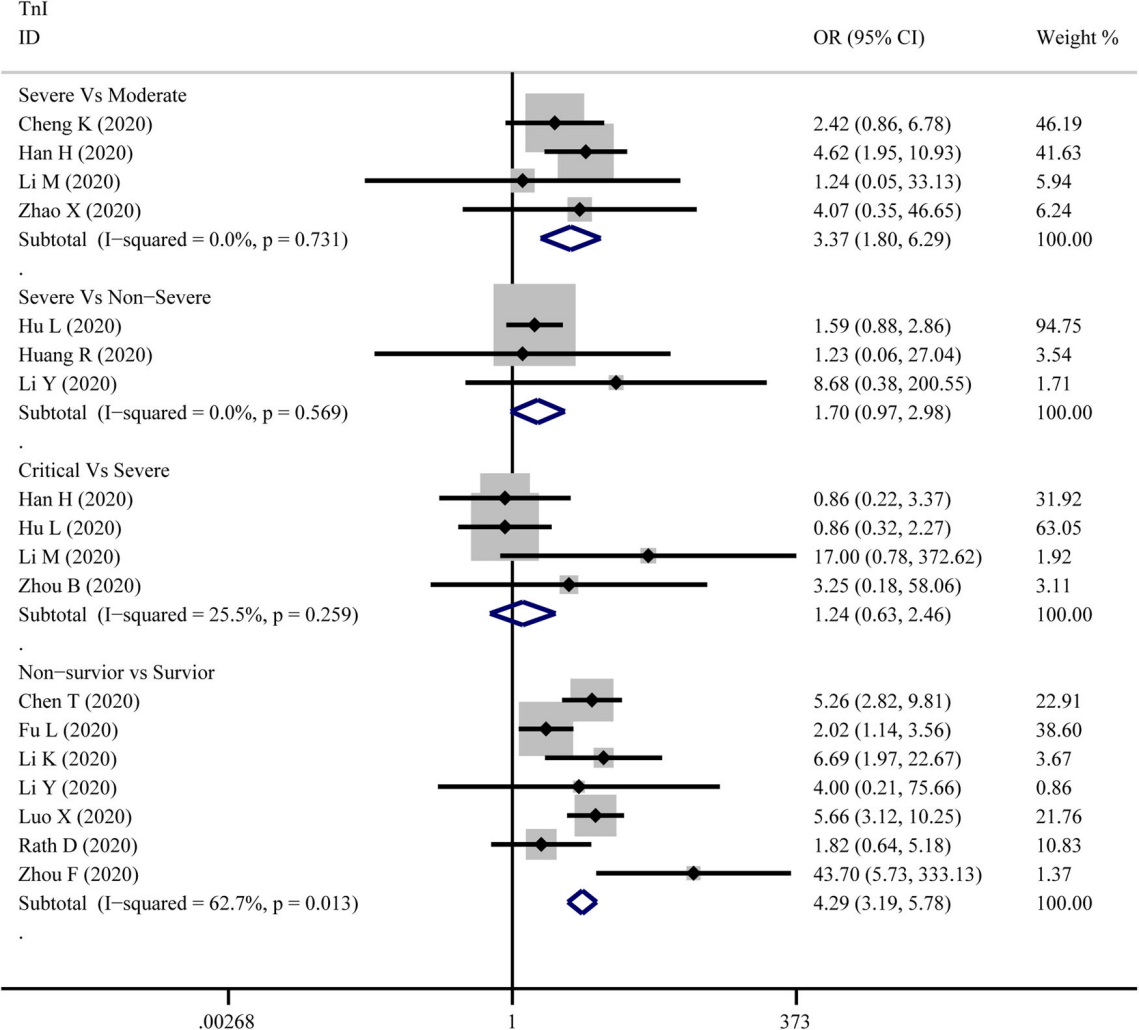
Forest plot comparisons of increasing troponin I (TnI) in patients with different severity of COVID‐19

**Figure 9:**
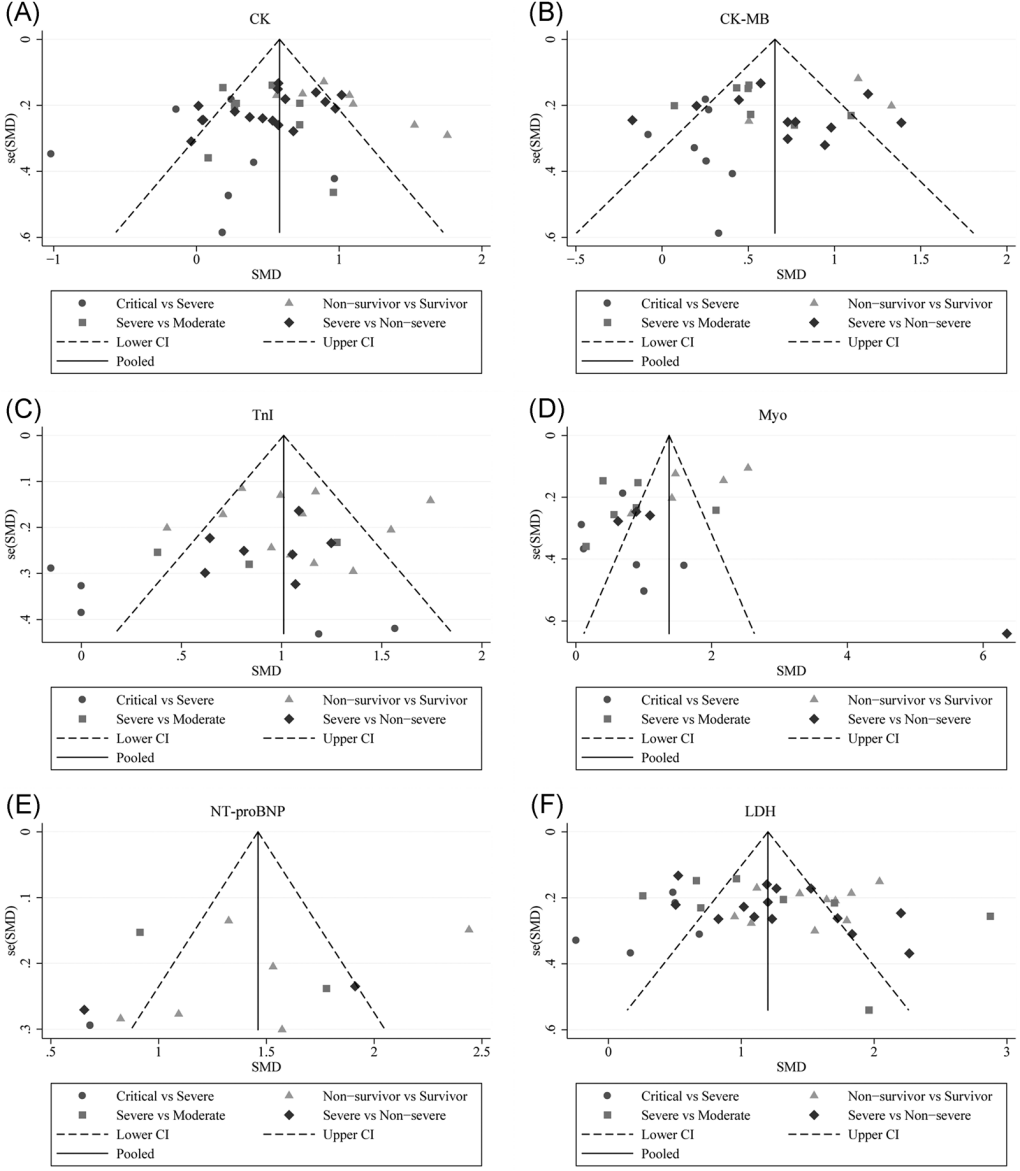
Funnel plot for the assessment of publication bias in continuous outcomes: (A) CK in different severity and outcome of COVID‐19, (B) CK‐MB in different severity and outcome of COVID‐19, (C) TnI in different severity and outcome of COVID‐19, (D) Myo in different severity and outcome of COVID‐19, (E) NT‐proBNP in different severity and outcome of COVID‐19, (F) LDH in different severity and outcome of COVID‐19

**Figure 10:**
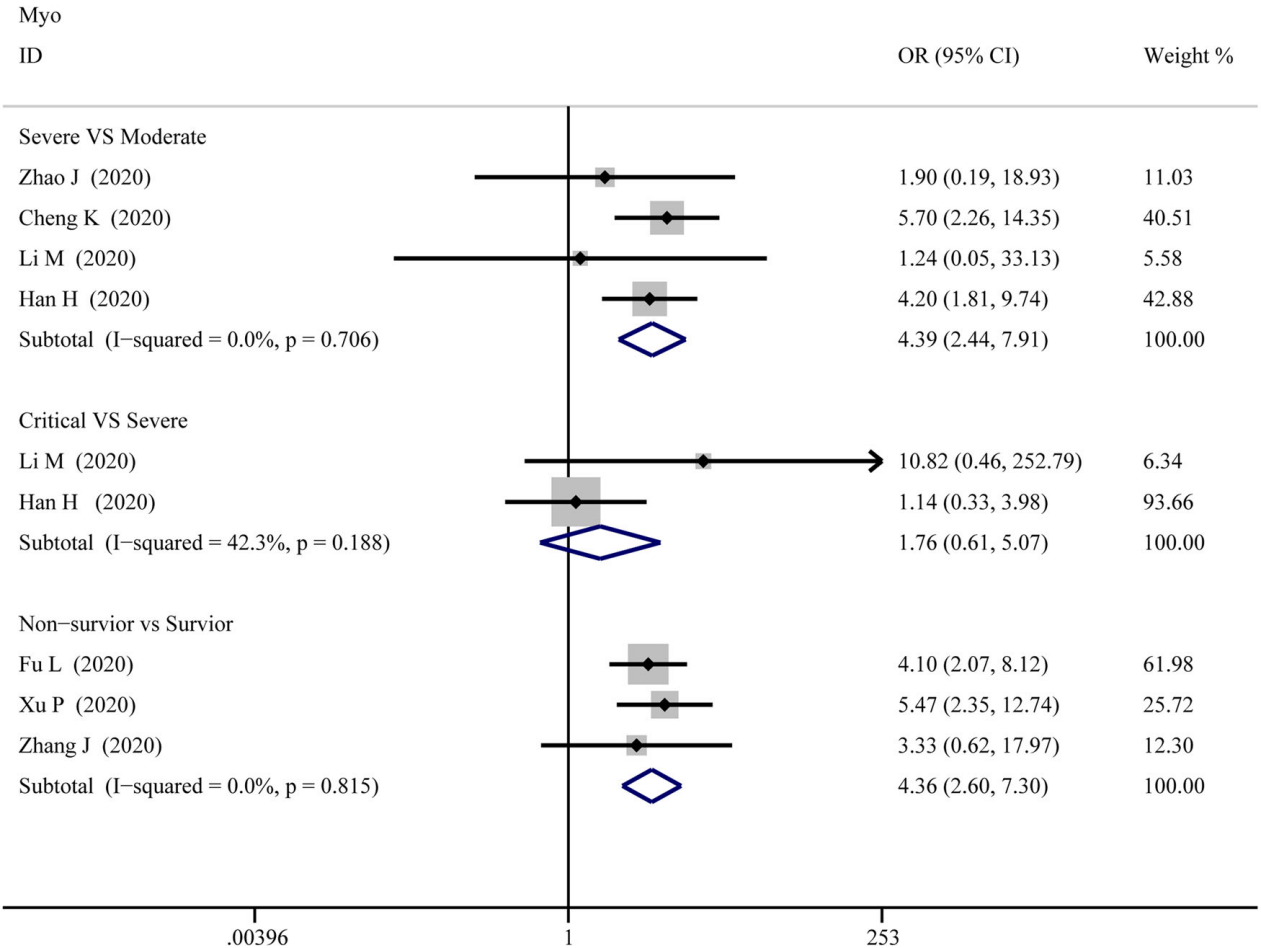
Forest plot comparisons of increasing myoglobin (Myo) in patients with different severity of COVID‐19

**Figure 11:**
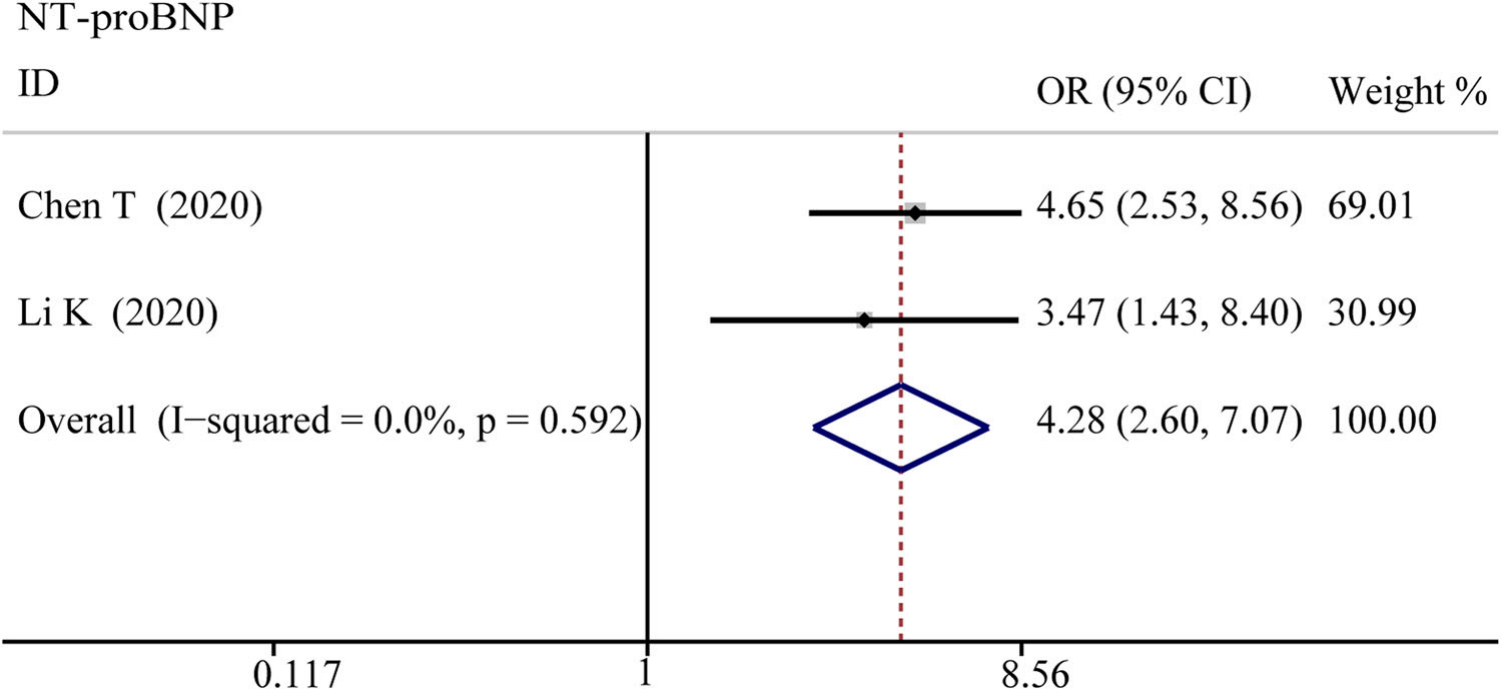
Forest plot comparisons of increasing N‐terminal pro‐b‐type natriuretic peptide (NT‐proBNP) in paitents with different severity of COVID‐19

1. CK
  The abnormal cases and increasing serum levels were all pooled for meta‐analysis. As can be seen from Figure 4, the combined OR were 2.66 (95% CI, 1.554–4.545; *p* = 0; *I* ^2^ = 0%) in the severe versus moderate group, 2.80 (95% CI, 1.86–4.23; *p* = 0; *I* ^2^ = 51.6%) in the severe versus non‐severe group after removing “Guan W 2020,” 2.683 (95% CI, 0.83–8.671; *p* = .106; *I* ^2^ = 0%) in critical versus severe group which means the number of the COVID‐19 patients with increased CK level was no different, and 2.42 (95% CI, 1.81–3.23; *p* = 0; *I* ^2^ = 0%) in the survivor versus non‐survivor group. In Figure 5, the SMD was showed similar trends that: 0.65 (95% CI, 0.27–1.04; *p* = .001; *I* ^2^ = 87.1%) in severe versus moderate group, 0.53 (95% CI, 0.37–0.68; *p* = 0; *I* ^2^ = 60.4%) in severe versus non‐severe group, and 1.19 (95% CI, 0.79–1.59; *p* = 0; *I* ^2^ = 91.7%) in survivor versus the non‐survivor group that higher serum levels of CK were observed in more severe cases except for critical versus severe group in which SMD is 0.09 (95% CI, −0.33 to 0.50; *p* = .685; *I* ^2^ = 65.2%). Sensitivity analysis by removing “Ling Y 2020,” “Li Q 2020” showed that overall estimates did not depend on a single publication in severe versus moderate (SMD = 0.42; 95% CI, 0.28–0.56; *p* = 0; *I* ^2^ = 32.7%) and survivor versus non‐survivor group (SMD = 1.04; 95% CI, 0.78–1.29; *p* = 0; *I* ^2^ = 71.1%) respectively. The funnel plot is presented in Figure 6. There were no publishing biases based on the results of Egger's test in the funnel plot.
2. CK‐MB
  Figure 7 compares the results obtained from the meta‐analysis of CK‐MB. The combined OR were 2.29 (95% CI, 1.25–4.19; *p* = .007; *I* ^2^ = 0%) in severe versus moderate group, 3.12 (95% CI, 1.78–5.47; *p* = 0; *I* ^2^ = 67.6%) in severe versus non‐severe group, and 7.37 (95% CI, 3.39–16.00; *p* = 0; *I* ^2^ = 0%) in survivor versus non‐survivor group which indicated a larger amount cases with higher CK‐MB were in more severe cases whereas no difference in critical versus severe group (OR = 2.26; 95% CI, 0.94–5.46; *p* = .069; *I* ^2^ = 0%). A significantly increased in serum level of CK‐MB was also observed in more severe COVID‐19 cases. The SMD of CK‐MB is the following in Figure 5: 0.53 (95% CI, 0.33–0.72; *p* = 0; *I* ^2^ = 51.8%) in severe versus moderate group, 0.60 (95% CI, 0.29–0.91; *p* = 0; *I* ^2^ = 85%) in severe versus non‐severe group, 0.37 (95% CI, 0.044–0.70; *p* = .026; *I* ^2^ = 55.4%) in critical versus severe group, and 1.49 (95% CI, 0.61–2.37; *p* = .00; *I* ^2^ = 96.9%) in survivor versus non‐survivor group. Sensitivity analysis by removing “Wang G 2020,” “Gao W 2020,” “Shi S 2020” showed that overall estimates did not depend on a single publication in severe versus non‐severe (SMD = 0.70; 95% CI, 0.44–0.96; *p* = 0; *I* ^2^ = 74.8%), critical versus severe (SMD = 0.22; 95% CI, 0.01–0.42; *p* = .039; *I* ^2^ = 0%) and survivor versus non‐survivor group (SMD = 1.10; 95% CI, 0.80–1.41; *p* = 0; *I* ^2^ = 64.1%), respectively. Based on the results of Egger's test, we found no evidence of publication bias in for CK‐MB in all groups (Figure 6).
3. TnI
  The combined OR of TnI were following in Figure 8 that 3.37 (95% CI, 1.80–6.292; *p* = 0; *I* ^2^ = 0%) in the severe versus moderate group, 1.70(95% CI, 0.97–2.98,*p* = .066; *I* ^2^ = 0%) in severe versus non‐severe group, 1.24 (95% CI, 0.63–2.46, *p* = .534; *I* ^2^ = 25.5%) in critical versus severe group, and 4.29 (95% CI, 3.19–5.78; *p* = 0; *I* ^2^ = 62.7%) in non‐survivor versus survivor group. The result of SMD obtained from critical versus severe group was 0.48 (95% CI, −0.183 to 1.14; *p* = .156; *I* ^2^ = 76.7%) displayed in Figure 5. The results of SMD showed that: 0.839 (95% CI, 0.308–1.371; *p* = .002; *I* ^2^ = 70.5%) in severe versus moderate group, 0.96 (95% CI, 0.784–1.13; *p* = 0; *I* ^2^ = 0.6%) in severe versus non‐severe group, and 1.13 (95% CI, 0.928–1.327; *p* = 0; *I* ^2^ = 76.9%) in survivor versus non‐survivor group after the sensitivity analysis by removing “Ma K 2020,” “Liu C 2020,” “Li Q 2020,” receptively, which means that higher serum levels of TnI were observed in more severe cases. According to the funnel plot in Figures 6 and 9 the results of Egger's test, there were no publish biases were observed.
4. Myo
  Figure 10 illustrates the patients of abnormal Myo in different severity The combined OR were 4.39 (95% CI, 2.44–7.91; *p* = 0; *I* ^2^ = 0%) in the severe versus moderate group, 4.36 (95% CI, 2.60–7.299; *p* = 0; *I* ^2^ = 0%) in the survivor versus non‐survivor group, which indicated larger amount cases with higher Myo were in more severe cases, while no difference was found in critical versus severe group (OR = 1.756; 95% CI, 0.608–5.071; *p* = .29; *I* ^2^ = 42.3%). As shown in Figure 5, the SMD of Myo are the following: 0.84 (95% CI, 0.36–1.32; *p* = .001; *I* ^2^ = 87.4%) in severe versus moderate group, 0.67 (95% CI, 0.25–1.086; *p* = .00; *I* ^2^ = 57.9%) in critical versus severe group, 2.09 (95% CI, 0.63–3.556; *p* = .005; *I* ^2^ = 95.8%) in severe versus non‐severe group, and 1.68 (95% CI, 1.23–2.13; *p* = 0; *I* ^2^ = 92.7%) in survivor versus non‐survivor group. Sensitivity analysis by removing “Li M 2020,” “Ling Y 2020,” and “Li Q 2020” showed that overall estimates did not depend on a single publication in severe versus moderate (SMD = 0.63; 95% CI, 0.35–0.91; *p* = 0; *I* ^2^ = 55.6%), severe versus non‐severe (SMD = 0.88; 95% CI, 0.59–1.17; *p* = 0; *I* ^2^ = 0%), and survivor versus non‐survivor group (SMD = 1.53; 95% CI, 1.18–1.88; *p* = 0; *I* ^2^ = 82.4%), respectively. The funnel plot is shown in Figures 6 and 9, respectively. Based on the results of Egger's test, we found no evidence of publication bias for Myo in all groups.
5. NT‐proBNP
  The combined OR was 4.285 (95% CI, 2.596–7.072; *p* = 0; *I* ^2^ = 0%) in the survivor versus non‐survivor group presented in Figure 11, which indicated a larger amount of cases with higher NT‐proBNP were in more severe cases. The serum level of NT‐proBNP significantly increased was also observed in more severe COVID‐19 cases. The SMD of NT‐proBNP are the following: 1.33 (95% CI, 0.48–2.17; *p* = .002; *I* ^2^ = 89.3%) in severe versus moderate group, 1.47 (95% CI, 0.71–2.22; *p* = 0; *I* ^2^ = 86%) in severe versus non‐severe group, and 1.50 (95% CI, 1.07–1.92; *p* = 0; *I* ^2^ = 87.2%) in survivor versus non‐survivor group. Sensitivity analysis by removing Shi S study showed that overall estimates did not depend on a single publication in survivor versus non‐survivor group (SMD = 1.35; 95% CI, 1.18–1.52; *p* = 0; *I* ^2^ = 22.3%). The results of the funnel plot are summarized in Figures 6 and 9, respectively. Based on the results of Egger's test, we found no evidence of publication bias in NT‐proBNP in all groups.
6. LDH

**Figure 12:**
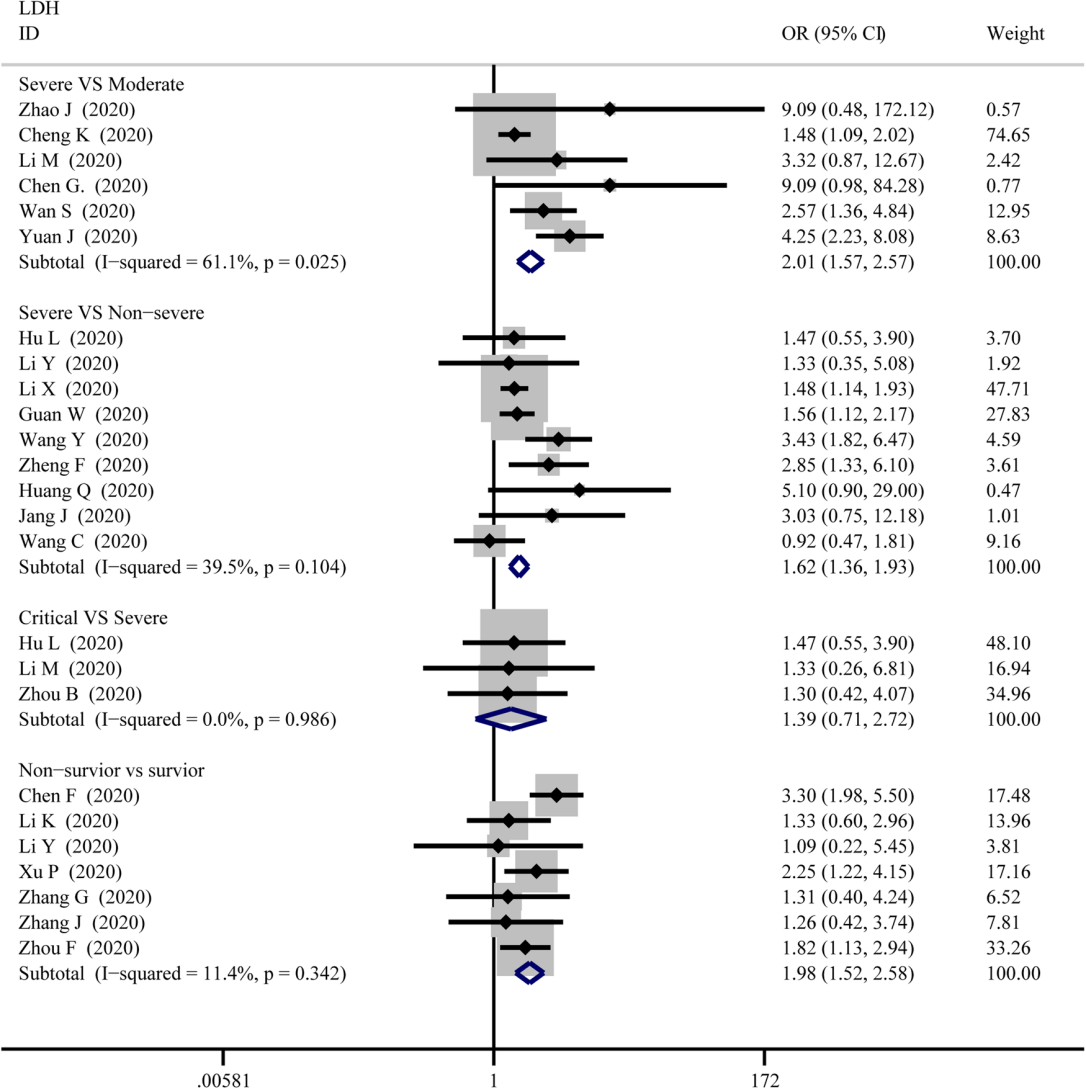
Forest plot comparisons of increasing lactate dehydrogenase (LDH) in patients with different severity of COVID‐19

As displayed in Figure 12, the combined OR were 2.01 (95% CI, 1.57–2.57; *p* = .025; *I* ^2^ = 61.1%) in the severe versus moderate group, 1.62 (95% CI, 1.36–1.93; *p* = 0; I^2^ = 39.5%) in the severe versus non‐severe group, and 1.98 (95% CI, 1.52–2.58; *p* = 0; *I* ^2^ = 11.4%) in survivor versus non‐survivor group, whereas, 1.39 (95% CI, 0.71–2.72; *p* = .34; *I* ^2^ = 0%) in the critical versus severe group which means there was no difference among the severity of COVID‐19 cases. The SMD of LDH was showed that higher serum levels of LDH were observed in severe cases (Figure 5). The results are following: 1.26 (95% CI, 0.75–1.78.; *p* = 0; *I* ^2^ = 92.3%) in the severe versus moderate group, 1.28 (95% CI, 1.01–1.56; *p* = 0; *I* ^2^ = 82.6%) in the severe versus non‐severe group, 0.40 (95% CI, 0.18–0.62; *p* = 0; *I* ^2^ = 27.8%) in the critical versus severe group, and 1.48 (95% CI, 1.24–1.72; *p* = 0; *I* ^2^ = 0.76%) in the survivor versus non‐survivor group. Sensitivity analysis by removing “Wu J 2020” showed that overall estimates did not depend on a single publication in severe versus non‐severe (SMD = 1.35; 95% CI, 1.10–1.59; *p* = 0; *I* ^2^ = 74.2%). There were no publishing biases based on the results of Egger's test in funnel plots (Figures 6 and 9).

**Figure 13:**
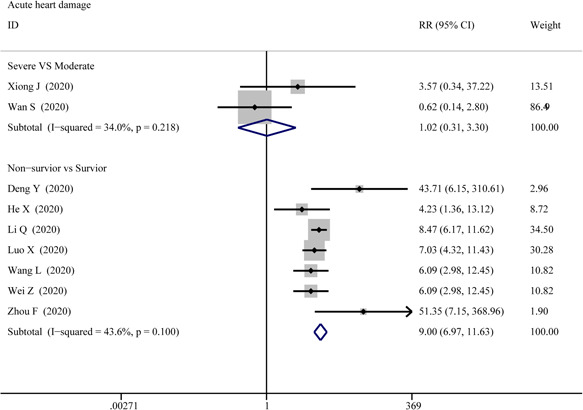
Forest plot comparisons of acute cardiac injury in patients with different severity of COVID‐19

#### 3.3.3 Acute cardiac injury

The data on acute cardiac injury among patients were extracted from 2 groups: the severe versus moderate group and the survivor versus non‐survivor group, then pooled respectively for meta‐analysis. The results are set out in Figure 12 that there were marked differences for acute cardiac injury between survivor versus non‐survivor cases (RR = 7.01; 95% CI, 5.64–8.71; *p* = 0; *I* ^2^ = 68.3%), while no differences between severe versus moderate cases (RR = 1.017; 95% CI, 1.017–0.313; *p* = .978; *I* ^2^ = 34%). Sensitivity analysis by removing “Chen T 2020” showed that overall estimates did not depend on a single publication in survivor versus non‐survivor group (RR = 9.005; 95% CI, 6.974–11.626; *p* = 0; *I* ^2^ = 43.6%). Based on the results of Egger's test, we found no evidence of publication bias for acute cardiac injury in all groups.

## 4 DISCUSSION

SARS‐CoV‐2, the third newly severe epidemic coronaviruses that have led to significant outbreaks and caused a big challenge after the SARS‐CoV occurred in 2002 and the MERS‐CoV that was identified in 2012.^3^ No pre‐existing immunity and definitive treatments can protect people, especially older adults and vulnerable members of the community, with prevalent comorbidities.^80^, ^81^, ^82^ Cardiac‐related comorbidities and myocardial damage have been identified in a non‐negligible number of COVID‐19 patients, and the interpretation of these features based on the COVID‐19's severity is essential for further interventions and therapeutics. This comprehensive meta‐analysis and systematic review mainly summarize the cardiac‐related complications, outcomes, and laboratory findings of 15,354 COVID‐19 cases from 70 studies. All subjects were classified into the severe versus moderate group, severe versus critical group, severe versus non‐severe group, and survivor versus non‐survivor group according to the selected articles, respectively. We found that a higher severity of COVID‐19 was associated with higher rates of hypertension, cardiovascular diseases, and acute cardiac injury in the four groups. Similarly, this clean pattern was also found in the number of COVID‐19 patients with increased serum levels of CK, CK‐MB, Myo, LDH, TnI, and NT‐proBNP, whereas no difference was witnessed in the severe versus critical group. Another important finding was compared with milder infection patients, those with a severe infection showed a significant increase in CK, CK‐MB, Myo, LDH, and TnI.

Various mechanisms can be suggested to explain our results in the meta‐analysis that patients suffering from hypertension or underlying cardiovascular diseases have a high risk of developing severe manifestations of COVID‐19. The mismatch of supply and demand of myocardial oxygen is a common phenomenon of severe viral diseases accompanied by insufficient systemic oxygenation during pneumonia in elderly patients with cardiovascular diseases and other chronic diseases.^82^ Another possible explanation for this might be that hypertension, cardiovascular diseases, and their treatments upregulate ACE2, especially with the use of RAAS inhibitors.^83^ As a membrane‐bound aminopeptidate receptor that expresses on epithelial cells, ACE2 converts the vasoconstrictor AngII which is hydrolyzed by ACE1 into the vasodilator angiotensin 1–7 (Ang1–7).^84^ ACE2 is higher expressed in the heart after receiving ARB or ACEI,^85^ interacts with SARS‐CoV‐2 spike protein, which leads to endothelial dysfunction and myocardial damage directly.^86^ Meanwhile, SARS‐CoV‐2 binding to ACE2 led to a reduction of the external ACE2 catalytic effect and replaced by internalization.^87^ Therefore, the possible downregulation of ACE2 and the subsequent increase of the pro‐inflammatory AngII together with the decrease of the cardioprotective Ang1–7 in patients with COVID‐19 may ultimately compromise heart function.^88^

The elevated serum levels of biomarkers of myocardial injury including CK, CK‐MB, LDH, TnI, and NT‐proBNP were strongly associated with severe forms of COVID‐19 from our research. Myocardial injury from SARS‐CoV‐2 infection involves many factors, and the mechanisms have not been fully elucidated yet. As mentioned previously, as a consequence of ACE2 expression in cardiac tissue, SARS‐CoV‐2 can directly cause heart damage. There have been studies showing that the heart is susceptible to SARS‐Cov‐2 infections.^89^ A further mechanism for explanations involves a cytokine storm, which is described as an excessive immune response towards SARS‐CoV‐2 infection.^90^, ^91^ Cytokines are essential to infection control, but when the immune system is deregulated including the imbalanced response of type 1 and type 2 T helper cells, a cytokine storm will be observed, resulting in an excessively elevated level of cytokines, such as C‐reactive protein interleukin 8 (IL‐8), IL‐10, procalcitonin, IL‐1b, and tumor necrosis factor‐α particularly IL‐6 coupled with tissue damage and multiple organ dysfunctions.^92^

There are several limitations that should be mentioned in our study. First, significant publication bias was observed in several results. A possible explanation is that some of the studies were published at the preprint server, which means some of the included studies are non‐peer‐reviewed scientific manuscripts. Secondly, as a novel infection disease, most of the including studies were retrospective case series, and no randomized controlled trial was included. The lack of RCT or prospective studies causes the consequence that it is hard to adjust for potentially confounding factors, and that might be a source of high heterogeneity. Third, different clinical laboratories used different ranges of normal values for laboratory indicators; therefore, we used SMD to value the relationship between increased serum level and severity of COVID‐19, which can achieve a definite ratio instead of numerical laboratory results. Finally, another limitation is that the majority of included studies were from China, only five of them are from other countries experiencing the COVID‐19 outbreak, which might lead to bias from races and geographic scope.

## 5 CONCLUSION

This meta‐analysis comprehensively investigates the differences in cardiac‐related comorbidities, outcomes, and laboratory indicators between patients with COVID‐19 who have different disease severity. The findings clearly indicate that hypertension, cardiovascular disease, acute cardiac injury, and related laboratory indicators are associated with the severity of COVID‐19. What is now needed are cross‐national prospectively designed observational or clinical trials that will help improve the certainty of the available evidence and treatment decisions for patients.

## CONFLICT OF INTERESTS

The authors declare that there are no conflict of interests.

## AUTHOR CONTRIBUTIONS

Zhengchuan Zhu and Miaoran Wang designed the study and wrote the manuscript. Qiaoyan Cai and Ling Zhang extracted the data and evaluated the RCTs quality. Jianfeng Chu, Jun Peng, and Keji Chen contributed to interpreting the results, draft reviewing, and finalizing the paper. All authors revised the report and approved the final version before submission.

## Data Availability

The data that support the findings of this study are available from the corresponding author upon reasonable request.
